# The First Report on the Transovarial Transmission of Microsporidian *Nosema bombycis* in Lepidopteran Crop Pests *Spodoptera litura* and *Helicoverpa armigera*

**DOI:** 10.3390/microorganisms9071442

**Published:** 2021-07-03

**Authors:** Boyan Pei, Chunxia Wang, Bin Yu, Dan Xia, Tian Li, Zeyang Zhou

**Affiliations:** 1 State Key Laboratory of Silkworm Genome Biology, Southwest University, Chongqing 400715, China; pby1149935728@126.com (B.P.); wangcx6164@outlook.com (C.W.); yubin5868@outlook.com (B.Y.); xdxdxdxjj@outlook.com (D.X.); 2 Chongqing Key Laboratory of Microsporidia Infection and Control, Southwest University, Chongqing 400715, China; 3 College of Life Science, Chongqing Normal University, Chongqing 400047, China

**Keywords:** microsporidia, *Nosema bombycis*, transovarial transmission, *Spodoptera litura*, *Helicoverpa armigera*

## Abstract

Microsporidia are ubiquitous fungi-related parasites infecting nearly all vertebrates and invertebrates. Microsporidian *Nosema bombycis* is a natural pathogen of multiple insects, including the silkworm and many agricultural and forest pests. *N. bombycis* can transovarially transmit in silkworm and cause huge economic losses to the sericulture. However, it remains unclear whether *N. bombycis* vertically transmits in the crop pests *Spodoptera litura* and *Helicoverpa armigera*. Here, we investigated the infection of *N. bombycis* in *S. litura* and *H. armigera* to illuminate its infectivity and transovarial transmission. In result, tissue examination with light microscopy revealed that the fat body, midgut, malpighian tubules, hemolymph, testis, and ovary were all infected in both pest pupae. Immunohistochemical analysis (IHA) of the ovariole showed that a large number of parasites in maturation and proliferation presented in follicle cell, nurse cell, and oocyte, suggesting that *N. bombycis* can infect and multiply in these cells and probably transovarially transmit to the next generations in both pests. Microscopic examination on the egg infection rate demonstrated that 50% and 38% of the *S. litura* and *H. armigera* eggs were congenitally infected, respectively. IHA of both eggs manifested numerous spores and proliferative pathogens in the oocyte, confirming that *N. bombycis* can invade into the female germ cell from the parent body. After hatching of the infected eggs, we detected the infection in offspring larvae and found large quantities of proliferative pathogens, confirming that *N. bombycis* can transovarially transmit in *S. litura* and *H. armigera*, and probably persists in both pest populations via congenital infection. In summary, our work, for the first time, proved that *N. bombycis* is able to vertically transmit in *S. litura* and *H. armigera* via infecting the oocyte in the parent, suggesting that *N. bombycis* could be a biological insecticide for controlling the population of crop pests.

## 1. Introduction

Microsporidia are a group of obligate intracellular parasites that infect nearly all vertebrates and invertebrates and are composed of over 200 genera and 1400 species [1]. Microsporidia have been found widely prevalent in insects, mammals, birds, and water [2,3]. Microsporidia not only cause human microsporidiosis [1,4] but also lead to huge economic losses by infecting economically important animals, such as silkworm, bee, shrimp, crab, and fish [5,6]. *Nosema bombycis* is the first identified microsporidia and causes silkworm pébrine disease. *N. bombycis* can horizontally and vertically transmit in silkworm [7] and is one of the biggest threats to sericulture [8]. In addition, *N. bombycis* is also a pathogen infecting multiple lepidopteran insects [9]. To date, over 1600 species of insects have been identified as Lepidoptera, many of which are agricultural and forest pests [10]. *Spodoptera litura* and *Helicoverpa armigera* are two typical lepidopteran pests and do great harm to plenty of economic crops, such as tomatoes, cotton, and maize [11,12]. It was reported that *Nosema spodopterae* isolated from *S. litura* and *H. armigera* showed a high virulence [13]. Yet, it is not known whether the *Nosema* can vertically transmit in *S. litura* and *H. armigera*.

The most common transmissive mode of most microsporidia is horizontal infection. However, for some microsporidia of arthropods, transovarial transmission is a key mode to maintain in the host population [14], especially for those that can only transmit vertically [15,16]. Except for *Encephalitozoon cuniculi* [17], the vertical transmission mode of most microsporidia usually refers to transovarial transmission. Microsporidian transovarial transmission occurs in two distinct ways, the most common of which is that the parasite enters the egg by infecting the ovary and related germ cells of the female host [18]. The other way is that the offspring of the infected female individuals eat the spores either in the yolk before and after hatching or sticking on the egg surface [19,20]. The complete process of transovarial transmission is composed of three major steps: the parasite first infects the ovariole of the female host, then enters into the mature eggs through oocytes, and continues to proliferate until the eggs develop into offspring larvae.

In this study, we characterized the infectivity of *N. bombycis* in tissues of *S. litura* and *H. armigera*, and scrupulously determined the transovarial transmission of the parasite in both crop pests.

## 2. Materials and Methods

### 2.1. Preparation of N. bombycis Spores

*N. bombycis* CQ1 (NbCQ1) was obtained from the State Key Laboratory of Silkworm Genome Biology and stored in the China Veterinary Microorganisms Collection and Management Center (CVCC No. 102059). Infected pupa tissues were homogenized in sterilized water and filtered using thick cotton in a 10 mL centrifuge tube. Spores were then purified by centrifugation at 10,000× *g* for 20 min in 90% Percoll. The purified spores were washed three times with sterilized water and then suspended in 0.5 mL water and finally stored at 4 °C for later use.

### 2.2. Rearing and Inoculating S. litura and H. armigera

The *S. litura* and *H. armigera* were purchased from Jiyuan Baiyun Industrial Co., Ltd., Henan, China. The larvae were reared with a fresh artificial diet at 26–28 °C and 70% relative humidity, 12 h light and dark, respectively. On the second day of the third instar, larvae were feed with 2 × 10^3^ NbCQ1 spores per larva. Adults were reared with 10% sucrose solution for energy supply. Eggs were hatched at 28 degrees in centigrade and 70% humidity.

### 2.3. Microscopic Observation of the Infections

The midgut, fat body, malpighian tubules, ovary, testis, and hemolymph of the infected pupae were collected and observed using OLYMPUS SZX16 with a 1× objective lens and a 10× eyepiece and photographed using OLYMPUS cellSens Standard 1.18. Tabletting tissues were directly observed using OLYMPUS BX53 with a 100× objective lens and a 10× eyepiece and photographed with cellSens Dimension 1.6.

### 2.4. Paraffin Section and Indirect Immunofluorescent Assay (IFA)

The normal and infected adult ovarioles on the 6th day after eclosion, eggs, and offspring larvae were prepared for paraffin section. The samples were fixed with 4% paraformaldehyde at 4 °C overnight, dehydrated with gradient ethanol (70%, 80%, and 90%) for 1 h each, then dehydrated with 95% and 100% ethanol twice for 30 min. After gradient dehydration, the slides were placed in the equivalent mixture of ethanol and xylene for 5 min and complete xylene for 3 min and then embedded by paraffin. The samples were sectioned into 4 μm slices and placed on the glass slides. After deparaffinization and hydration, the slices were incubated with antigen repair solution and stored at 98–100 °C for 20 min, and then incubated with polyclonal antibody against NbCQ1 at room temperature for 90 min and washed three times with PBST (0.01 M PBS + 0.05% Tween 20) each for 5 min, followed by incubation with goat anti-rabbit secondary antibody labeled with Dylight 488, Calcofluor White M2R for staining chitin, and propidium Iodide for staining nucleus in the dark for 40 min. The slides were suspended by Fluoromount Aqueous Mounting Medium (Sigma (Shanghai, China), F4680) and mounted with a cover glass after washing 3 times with PBST [21]. The samples were observed and photographed using the OLYMPUS Biological Confocal Laser Scanning Microscope FV1200 with 200× and 1000× magnifications for global and local observations, respectively.

### 2.5. PCR Amplification

Insect eggs were collected for extracting genomic DNA (gDNA) using the E.Z.N.A. Tissue DNA Kit according to the manufacturer’s instructions. PCR amplification with the primers targeted the large subunit ribosomal DNA (LSU, Forward primer: 5′-GGGGAAAGAAGACCCTGT-3′, Reverse primer: 5′-TCTGTCACCTCCAATCAA-3′) of NbCQ1. The amplification system was composed of 12.5 µL PrimeSTAR premix DNA polymerase (TaKaRa, R045Q), 0.4 µM primers, 1 µL genomic DNA extraction, and water up to 25 µL. Amplification was performed as the following procedures: pre-denaturation at 98 °C for 2 min, denaturation at 98 °C for 15 s, annealing at 62 °C for 15 s, extension for 35 cycles at 72 °C each for 10 s, and final extension at 72 °C for 10 min. An aliquot of 10μL from each PCR product was run on a 1.5% agarose gel to visualize the PCR product.

## 3. Results

### 3.1. NbCQ1 Infection in the Pupa of S. litura and H. armigera

**Figure 1:**
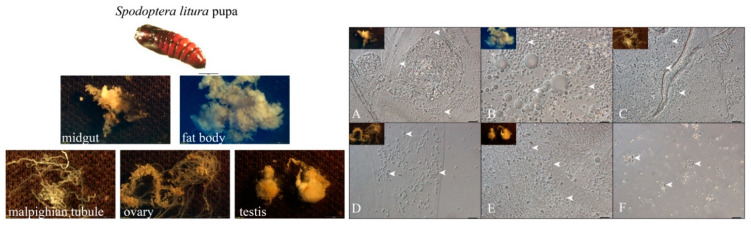
Microscopy observation of NbCQ1 infections in *S. litura* pupa tissues. (**A**) midgut, (**B**) fat body, (**C**) malpighian tubules, (**D**) ovary, (**E**) testis, (**F**) hemolymph. The bar indicates 10 μm. The arrowhead shows the NbCQ1 spore.

**Figure 2:**
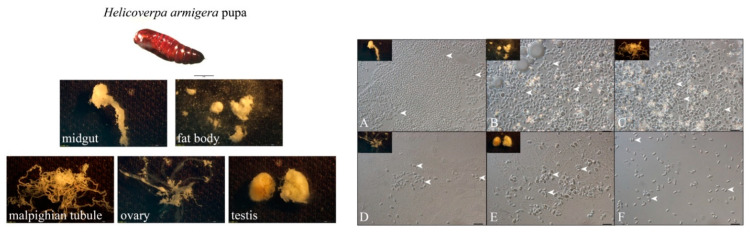
Microscopic observation of NbCQ1 infections in *H. armigera* pupa tissues. (**A**) midgut, (**B**) fat body, (**C**) malpighian tubules, (**D**) ovary, (**E**) testis, (**F**) hemolymph. The bar indicates 10 μm. The arrowhead shows the NbCQ1 spore.

The infection of NbCQ1 in pupa tissues of the *S. litura* (Figure 1) and *H. armigera* (Figure 2) was observed using a light microscope. As a result, all checked tissues of both animals were infected, including the midgut (Figure 1A and Figure 2A), fat body (Figure 1B and Figure 2B), malpighian tubules (Figure 1C and Figure 2C), ovary (Figure 1D and Figure 2D), testis (Figure 1E and Figure 2E), and hemolymph (Figure 1F and Figure 2F). The fat body and midgut were infected most heavily.

### 3.2. NbCQ1 Infection in the Ovariole of S. litura and H. armigera

**Figure 3:**
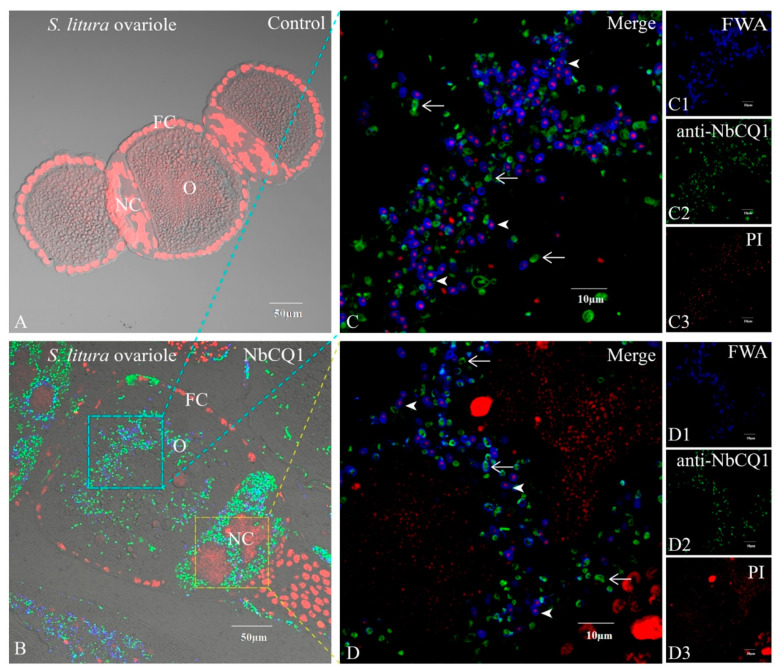
Paraffin sections of *S. litura* ovariole infected by NbCQ1. (**A**) Uninfected ovariole; (**B**) Infected ovariole; (**C**) An enlarged view of the infection of oocyte; (**D**) An enlarged view of the infection of follicle cell; (**C1**,**D1**) NbCQ1 spores stained with FWA; (**C2**,**D2**) Proliferative NbCQ1 labeled with antibody; (**C3**,**D3**) Nucleus stained by PI. FC: follicle cell; O: oocyte; NC: nurse cell; Arrowhead shows mature spore; Arrow shows parasite proliferation.

**Figure 4:**
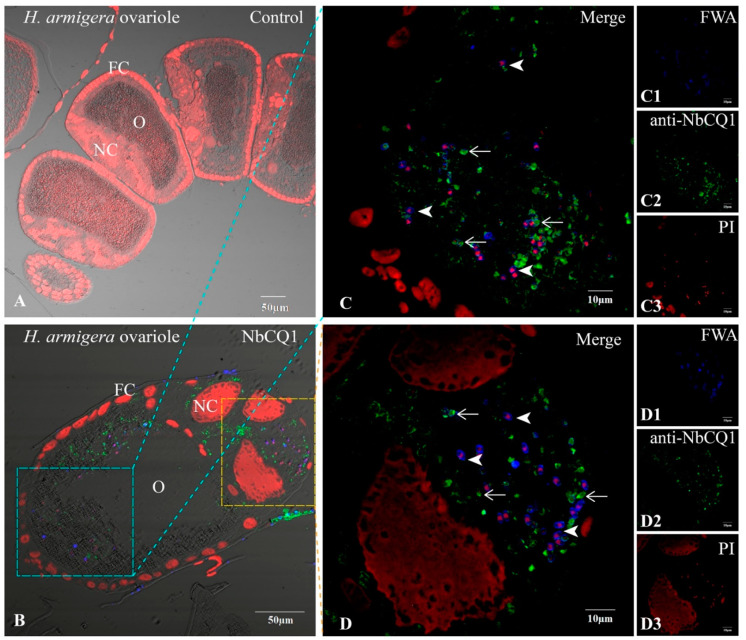
Paraffin sections of *H. armigera* ovariole infected by NbCQ1. (**A**) Uninfected ovariole; (**B**) Infected ovariole; (**C**) An enlarged view of the infection of oocyte; (**D**) An enlarged view of the infection of follicle cell; (**C1**,**D1**) NbCQ1 spores stained with FWA; (**C2**,**D2**) Proliferative NbCQ1 labeled with antibody; (**C3**,**D3**) Nucleus stained by PI. FC: follicle cell; O: oocyte; NC: nurse cell; Arrowhead shows mature spores; Arrow shows parasites in proliferation.

No infectious sign was found in the normal ovariole tissues (Figure 3A and Figure 4A), but a large number of parasites were observed in the infected ovarioles of *S. litura* and *H. armigera*, respectively (Figure 3B and Figure 4B). Mature spores and meronts were found in the oocytes (Figure 3C and Figure 4C), nurse cells, and follicle cells, among which the nurse cells were most heavily infected and full of pathogens in multiple stages (Figure 3D and Figure 4D).

### 3.3. NbCQ1 Infection in the Egg of S. litura and H. armigera

**Figure 5:**
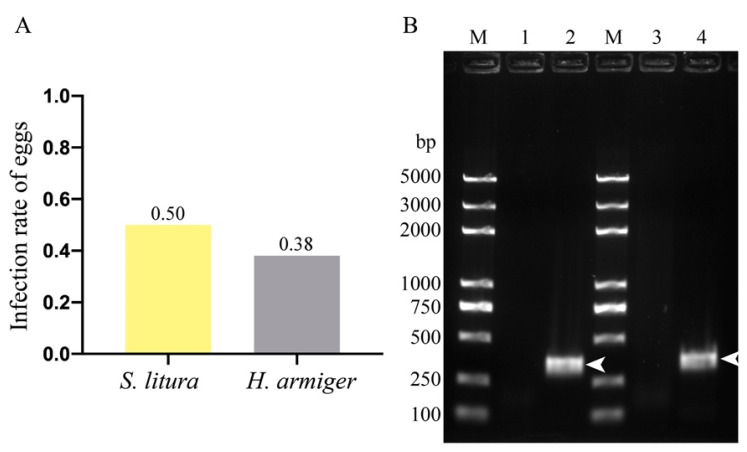
Determination of NbCQ1 infection in the eggs of *S. litura* and *H. armigera*. (**A**) Infection rates of NbCQ1 in the eggs of *S. litura* and *H. armigera* were counted by light-microscopic observation. (**B**) Infections of NbCQ1 in the eggs of *S. litura* and *H. armigera* were detected using PCR with primers targeting NbCQ1 LSU. PCR products were verified using the agarose gel electrophoresis. (Lane M) Trans2k Plus DNA Marker; (Lane 1, 3) PCR amplification of normal *S. litura* and *H. armigera* egg gDNA; (Lane 2, 4) PCR amplification of infected *S. litura* and *H. armigera* egg gDNA; Arrowheads showed the PCR products of NbCQ1 LSU.

**Figure 6:**
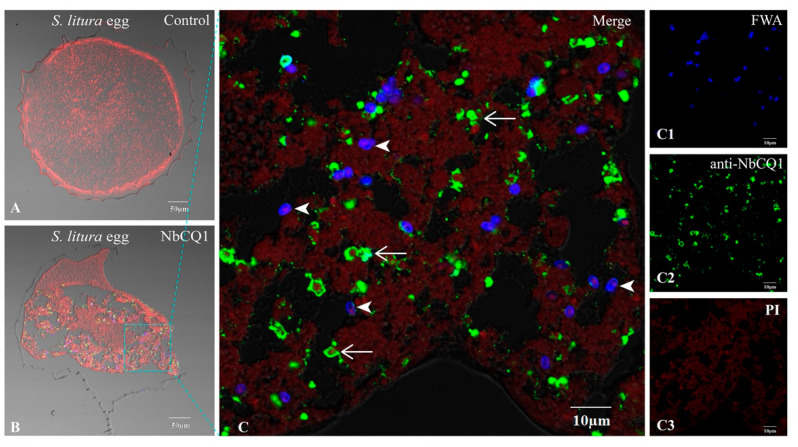

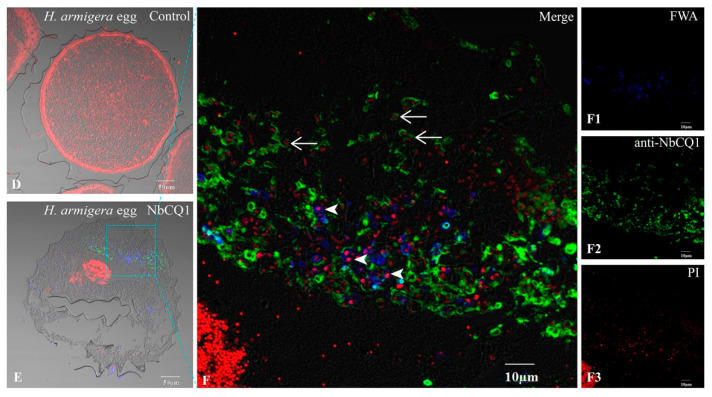
Paraffin sections of *S. litura* and *H. armigera* eggs infected by NbCQ1. (**A**) Uninfected *S. litura* egg; (**B**) NbCQ1-infected *S. litura* egg; (**C**) An enlarged view of NbCQ1 infections in the oocytes of *S. litura* egg; (**C1**) NbCQ1 spores stained with FWA; (**C2**) Proliferative NbCQ1 labelled with antibody; (**C3**) Nucleus stained with PI. The arrowhead shows NbCQ1 spores; the arrow shows proliferative pathogens. (**D**) Uninfected *H. armigera* egg; (**E**) NbCQ1-infected *S. litura* egg; (**F**) An enlarged view of NbCQ1 infections in the oocytes of *S. litura* egg; (**F1**) NbCQ1 spores stained with FWA; (**F2**) Proliferative NbCQ1 labelled with antibody; (**F3**) Nucleus stained with PI. The arrowhead shows NbCQ1 spores; the arrow shows proliferative pathogens.

We first counted the infection rate of eggs using a light microscope and found that 50% (25/50) and 38% (19/50) eggs of *S. litura* and *H. armigera* were infected, respectively (Figure 5A). We then confirmed the egg infection using PCR with specific primers targeting NbCQ1 LSU (Figure 5B). Furthermore, we analyzed the paraffin sections of infected eggs using IFA to characterize the pathogens inside (Figure 6). As a result, a large number of mature spores stained with FWA in blue and proliferative parasites labeled with antibodies in green were observed in the egg oocytes of *S. litura* (Figure 6A–C) and *H. armigera* (Figure 6D–F), respectively.

### 3.4. NbCQ1 Infection in the Offspring Larva of S. litura and H. armigera

**Figure 7:**
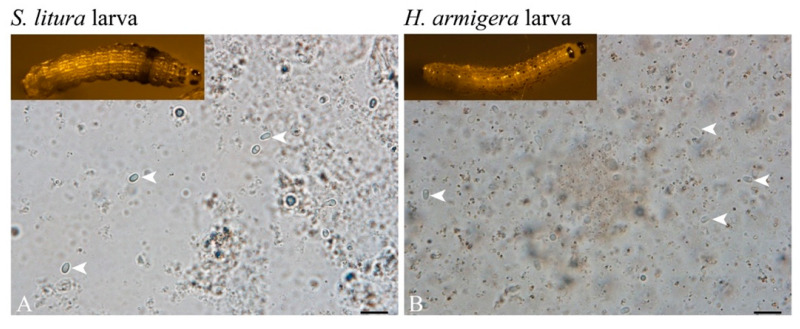
Microscopic observation on the tissue of offspring larvae of *S. litura* and *H. armigera*. (**A**) *S. litura* offspring larvae; (**B**) *H. armigera* offspring larvae. All bars are 10 μm, arrowhead shows the NbCQ1 spore.

**Figure 8:**
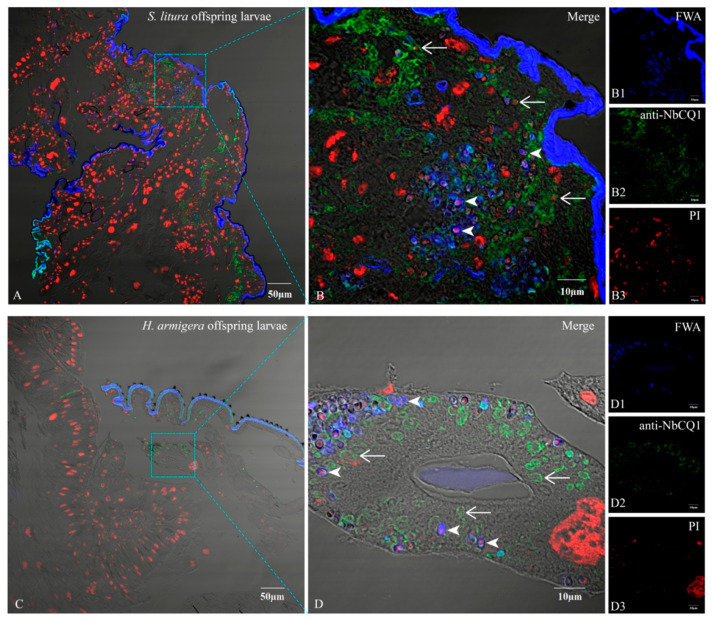
Paraffin section of the offspring larvae tissue of *S. litura* and *H. armigera*. (**A**) The tissues of *S. litura* offspring larvae infected by NbCQ1; (**B**) An enlarged view of NbCQ1 infections in *S. litura* offspring larva; (**B1**) NbCQ1 spores stained with FWA; (**B2**) Proliferative NbCQ1 labeled with antibody; (**B3**) Nucleus stained by PI. (**C**) The tissues of *H. armigera* offspring larvae infected by NbCQ1; (**D**) An enlarged view of NbCQ1 infections in *H. armigera* offspring larva; (**D1**) NbCQ1 spores stained with FWA; (**D2**) Proliferative NbCQ1 labeled with antibody; (**D3**) Nucleus stained by PI. The arrowhead shows the NbCQ1 spores, and the arrow shows pathogens in proliferation.

Tissues of the *S. litura* and *H. armigera* offspring larvae 7 days after hatching were dissected to verify the infection with a light microscope and paraffin section. Mature spores were observed in the offspring larva of *S. litura* and *H. armigera*, respectively (Figure 7). The paraffin section analysis showed that some parasites in maturation and proliferation were present in both larvae (Figure 8).

## 4. Discussion

Microsporidian infection and proliferation were observed in the *S. litura and H. armigera* ovaries, ovarioles, eggs, and offspring, which implies a transovarial transmission of the infection in both pests.

The *N. bombycis* infection and transmission have been well characterized in silkworm, but little is known about its infection in crop pests. Our work, for the first time, reported the infectivity and transovarial transmission of *N. bombycis* in *S. litura* and *H. armigera*, providing a promising biological insecticide to control these seriously harmful crop pests. However, before developing *N. bombycis* to a biotic pesticide, the mortality rates of individuals and impacts on pest populations should be determined and evaluated. Furthermore, the effect on non-target entomofauna must be investigated in multiple ecological systems, which could be a limit to the widespread use of insecticides [22,23,24]. Therefore, much more work remains to figure out the lethality and transmission of *N. bombycis* in these pests and other insects, although it is never easy to evaluate the virulence of microsporidia when facing a complicated environment, such as natural enemies and pesticides [25].

Chemical insecticides have been widely used to control *S. litura* and *H. armigera*, for example, chlorantraniliprole, organochlorines, and pyrethroids [26,27]. However, besides the environmental damage, the intensive use of these insecticides has caused a dramatic increase in resistant pest variants [28]. Instead, the biological reagents are now considered the best way to control pests, considering the side effects of the chemicals on both environment and human health [29]. Microsporidia are thought to be one of the ideal agents. For example, *Antonospora locustae* have been widely used as a microbial insecticide to control grasshopper [30]. Some other microsporidia, such as *Nosema pyrausta* and *Nosema lymantriae*, may also be used in pest control [31,32].

It was reported that even if one species of microsporidia may have a variety of hosts, yet they can only vertically transmit in their specific host, such as *Edhazardia aedis* in *Aedes aegypti* and *Amblyospora connecticus* in *Aedes cantator* [33,34]. However, *N. bombycis* can transovarially transmit in multiple insects, raising an interesting question about what is the mechanism that determines its high effective transmission. Previous studies showed that microsporidia localized around or contacted with yolk in host oocyte [35,36]. The vitellus is a fundamental nutrient for oocyte and formed by absorbing and accumulating vitellogenin, which has been found to play vital roles during the transovarial transmission of begomoviruses and *Wolbachia* [37,38,39]. These studies provide important clues to figure out how *N. bombycis* transovarially transmit using the infection models of *B. mori*, *S. litura,* and *H. armiger*.

## 5. Conclusions

*N. bombycis* can vertically transmit in *S. litura* and *H. armigera* by invading the female ovariole and oocyte. The horizontal and transovarial transmission of *N. bombycis* in both pests suggest its potential as a biocontrol agent and provide a good model for understanding the mechanisms of microsporidian vertical transmission.

## Data Availability

The data that support the findings of this study are available from the corresponding author upon reasonable request.

## References

[1] Fayer R., Santin-Duran M., Weiss L.M., Becnel J.J. (2014). Epidemiology of Microsporidia in Human Infections. Microsporidia Pathogens of Opportunity.

[2] Ruan Y., Xu X., He Q., Li L., Guo J., Bao J., Pan G., Li T., Zhou Z. (2021). The largest meta-analysis on the global prevalence of microsporidia in mammals, avian and water provides insights into the epidemic features of these ubiquitous pathogens. Parasites Vectors.

[3] Han B., Weiss L.M. (2017). Microsporidia: Obligate Intracellular Pathogens Within the Fungal Kingdom. Microbiol. Spectr..

[4] Oguz Kaya I., Dogruman Al F., Mumcuoglu I. (2018). Investigation of Microsporidia prevalence with calcofluor white and uvitex 2B chemiluminescence staining methods and molecular analysis of species in diarrheal patients. Mikrobiyol. Bul..

[5] Joseph J., Sharma S., Murthy S.I., Krishna P.V., Garg P., Nutheti R., Kenneth J., Balasubramanian D. (2006). Microsporidial keratitis in India: 16S rRNA gene-based PCR assay for diagnosis and species identification of microsporidia in clinical samples. Investig. Ophthalmol. Vis. Sci..

[6] Franzen C., Muller A. (1999). Molecular techniques for detection, species differentiation, and phylogenetic analysis of microsporidia. Clin. Microbiol. Rev..

[7] Han M.-S., Watanabe H. (1988). Transovarian transmission of two microsporidia in the silkworm *Bombyx mori* and disease occurrence in the progeny popultion. J. Invertebr. Pathol..

[8] Pan G., Xu J., Li T., Xia Q., Liu S.L., Zhang G., Li S., Li C., Liu H., Yang L. (2013). Comparative genomics of parasitic silkworm microsporidia reveal an association between genome expansion and host adaptation. BMC Genom..

[9] Kashkarova L.F., Khakhanov A.I. (1980). Range of the hosts of the causative agent of pebrine (*Nosema bombycis*) in the mulberry silkworm. Parazitologiia.

[10] Mereghetti V., Chouaia B., Montagna M. (2017). New Insights into the Microbiota of Moth Pests. Int. J. Mol. Sci..

[11] Figueiredo E., Goncalves C., Duarte S., Godinho M.C., Mexia A., Torres L. (2020). Risk Assessment for Tomato Fruitworm in Processing Tomato Crop-Egg Location and Sequential Sampling. Insects.

[12] Bragard C., Dehnen-Schmutz K., Di Serio F., Gonthier P., Jacques M.-A., Jaques Miret J.A., Justesen A.F., Magnusson C.S., Milonas P., EFSA Panel on Plant Health (PLH) (2019). Pest categorisation of Spodoptera litura. EFSA J..

[13] Tsai S.J., Lo C.F., Soichi Y., Wang C.H. (2003). The characterization of microsporidian isolates (Nosematidae: Nosema) from five important lepidopteran pests in Taiwan. J. Invertebr. Pathol..

[14] Dunn A.M., Terry R.S., Smith J.E. (2001). Transovarial transmission in the microsporidia. Adv. Parasitol..

[15] Terry R.S., Dunn A.M., Smith J.E. (1997). Cellular distribution of a feminizing microsporidian parasite: A strategy for transovarial transmission. Parasitology.

[16] Ni X., Backus E.A., Maddox J.V. (1997). Transmission Mechanisms of *Nosema empoascae* (Microspora: Nosematidae) in *Empoasca fabae* (Homoptera: Cicadellidae). J. Invertebr. Pathol..

[17] Kotkova M., Sak B., Hlaskova L., Kvetonova D., Kvac M. (2018). Evidence of transplacental transmission of *Encephalitozoon cuniculi* genotype II in murine model. Exp. Parasitol..

[18] Kellen W.R., Lindegren J.E. (1973). Transovarian transmission of *Nosema plodiae* in the Indian-meal moth, *Plodia interpunctella*. J. Invertebr. Pathol..

[19] Vavra J., Undeen A.H. (1970). *Nosema algerae* n. sp. (Cnidospora, Microsporida) a pathogen in a laboratory colony of *Anopheles stephensi* Liston (Diptera, Culicidae). J. Protozool..

[20] Canning E.U. (1982). An evaluation of protozoal characteristics in relation to biological control of pests. Parasitology.

[21] Chen J., Geng L., Long M., Li T., Li Z., Yang D., Ma C., Wu H., Ma Z., Li C. (2013). Identification of a novel chitin-binding spore wall protein (NbSWP12) with a BAR-2 domain from *Nosema bombycis* (microsporidia). Parasitology.

[22] De Araujo-Coutinho C.J., Cunha Ade B., Serra-Freire N.M., de Mello R.P. (2003). Evaluation of the impact of *Bacillus thuringiensis* serovar israelensis and Temephos, used for the control of Simulium (Chirostilbia) pertinax Kollar, 1832 (Diptera, Simuliidae) on the associated entomofauna, Paraty, state of Rio de Janeiro, Brazil. Mem. Inst. Oswaldo Cruz.

[23] Jurc M., Hauptman T., Pavlin R., Borkovič D. (2016). Target and non-target beetles in semiochemical-baited cross vane funnel traps used in monitoring *Bursaphelenchus xylophilus* (PWN) vectors in pine stands. Phytoparasitica.

[24] Miles M., Kemmitt G., Bakker F., Aldershof S. (2008). The impact of mancozeb on entomofauna communities in apple orchards. Commun. Agric. Appl. Biol. Sci..

[25] Shapiro-Ilan D.I., Bruck D.J., Lacey L.A., Vega F.E., Kaya H.K. (2012). Chapter 3—Principles of Epizootiology and Microbial Control. Insect Pathology.

[26] Kong F., Song Y., Zhang Q., Wang Z., Liu Y. (2021). Sublethal Effects of Chlorantraniliprole on *Spodoptera litura* (Lepidoptera: Noctuidae) Moth: Implication for Attract-And-Kill Strategy. Toxics.

[27] Saleem M.A., Ahmad M., Ahmad M., Aslam M., Sayyed A.H. (2008). Resistance to selected organochlorin, organophosphate, carbamate and pyrethroid, in *Spodoptera litura* (Lepidoptera: Noctuidae) from Pakistan. J. Econ. Entomol..

[28] Tian L., Gao X., Zhang S., Zhang Y., Ma D., Cui J. (2021). Dynamic changes of transcriptome of fifth-instar spodoptera litura larvae in response to insecticide. 3 Biotech.

[29] Pascale A., Laborde A. (2020). Impact of pesticide exposure in childhood. Rev. Environ. Health.

[30] Slamovits C.H., Williams B.A., Keeling P.J. (2004). Transfer of *Nosema locustae* (Microsporidia) to *Antonospora locustae* n. comb. based on molecular and ultrastructural data. J. Eukaryot. Microbiol..

[31] Lewis L.C., Bruck D.J., Prasifka J.R., Raun E.S. (2009). *Nosema pyrausta*: Its biology, history, and potential role in a landscape of transgenic insecticidal crops. Biol. Control.

[32] Solter L.F., Hajek A.E., Hajek A.E., Glare T.R., O’Callaghan M. (2009). Control of Gypsy Moth, *Lymantria dispar*, in North America since 1878. Use of Microbes for Control and Eradication of Invasive Arthropods.

[33] Andreadis T.G. (1989). Host specificity of *Amblyospora connecticus* (Microsporida: Amblyosporidae), a polymorphic microsporidian parasite of Aedes cantator (Diptera: Culicidae). J. Med. Entomol..

[34] Becnel J.J., Johnson M.A. (1993). Mosquito host range and specificity of *Edhazardia aedis* (Microspora: Culicosporidae). J. Am. Mosq. Control Assoc..

[35] Nordin G.L. (1975). Transovarial transmission of a *Nosema* sp. infecting *Malacosoma americanum*. J. Invertebr. Pathol..

[36] Raina S.K., Das S., Rai M.M., Khurad A.M. (1995). Transovarial transmission of *Nosema locustae* (Microsporida: Nosematidae) in the migratory locust *Locusta migratoria* migratorioides. Parasitol. Res..

[37] Huo Y., Liu W., Zhang F., Chen X., Li L., Liu Q., Zhou Y., Wei T., Fang R., Wang X. (2014). Transovarial transmission of a plant virus is mediated by vitellogenin of its insect vector. PLoS Pathog..

[38] Guo Y., Hoffmann A.A., Xu X.Q., Mo P.W., Huang H.J., Gong J.T., Ju J.F., Hong X.Y. (2018). Vertical Transmission of *Wolbachia* Is Associated With Host Vitellogenin in *Laodelphax striatellus*. Front. Microbiol..

[39] Wei J., He Y.Z., Guo Q., Guo T., Liu Y.Q., Zhou X.P., Liu S.S., Wang X.W. (2017). Vector development and vitellogenin determine the transovarial transmission of begomoviruses. Proc. Natl. Acad. Sci. USA.

